# New-onset adrenal insufficiency in COVID-19 patients without preexisting adrenal disease: A systematic review

**DOI:** 10.5339/qmj.2026.35

**Published:** 2026-06-15

**Authors:** Iman Fatima, Muneeb Ali Saif, Assylzhan M. Messova, Sagira T. Abdrahmanova, Nadiar M. Mussin, Amin Tamadon

**Affiliations:** 1 Pediatric Diseases Department, Astana Medical University, Astana, Kazakhstan; 2 Department of General Surgery, West Kazakhstan Marat Ospanov Medical University, Aktobe, Kazakhstan; 3 Department of Natural Sciences, West Kazakhstan Marat Ospanov Medical University, Aktobe, Kazakhstan

**Keywords:** COVID-19, SARS-CoV-2, adrenal insufficiency, systematic review, post-acute COVID-19 syndrome

## Abstract

**Introduction:**

Coronavirus disease 2019 (COVID-19) is increasingly recognized as a multisystem disorder with potential endocrine sequelae. Emerging reports suggest that adrenal insufficiency (AI) may occur in patients with COVID-19 without preexisting adrenal disease, but the clinical spectrum and strength of evidence remain unclear. To systematically review the literature on new-onset AI associated with COVID-19 in patients without prior adrenal disorders, focusing on clinical presentation, diagnostic approaches, and underlying mechanisms.

**Methods:**

A systematic review was conducted in accordance with Preferred Reporting Items for Systematic Reviews and Meta-Analyses (PRISMA) 2020 guidelines. PubMed/MEDLINE, Scopus, and Web of Science were searched from inception to December 28, 2025. Eligible studies included observational studies, case reports, and case series reporting AI in COVID-19 patients without known adrenal disease. Risk of bias was assessed using the Newcastle–Ottawa Scale and Joanna Briggs Institute tools. Given the heterogeneity, a qualitative synthesis was conducted.

**Results:**

Nine primary studies (two observational studies and seven case reports) were included. Four narrative reviews were used solely for contextual discussion. Cases involved adults and children and occurred during acute infection or the post-COVID period. Most reports described biochemically confirmed central AI, while fewer reported primary AI due to adrenal infarction, hemorrhage, or autoimmune adrenalitis. Some presentations based mainly on steroid responsiveness were interpreted as probable critical illness–related corticosteroid insufficiency. Diagnostic thresholds and the use of ACTH stimulation testing varied. Most patients improved with glucocorticoid replacement.

**Conclusion:**

New-onset AI has been reported in temporal association with COVID-19 and may represent an infrequently reported but clinically significant endocrine manifestation. Prospective studies with standardized hormonal assessment are needed to clarify incidence, mechanisms, and screening strategies.

**Registration:**

The review was registered with PROSPERO under registration number CRD420251275141.

## 1. INTRODUCTION

Adrenal insufficiency (AI) is a potentially fatal endocrine disorder caused by the lack of secretion of glucocorticoid and/or mineralocorticoid. It is categorized as primary (adrenal cortex destruction), secondary (pituitary adrenocorticotropic hormone (ACTH) deficiency), or tertiary (hypothalamic corticotropin-releasing hormone (CRH) deficiency).^1^ Autoimmune adrenalitis in adults is associated with >70% of primary AI cases, with the rest of the cases including tuberculosis, malignant infiltration, adrenal hemorrhage, and iatrogenic autoimmune checkpoint inhibitors and bilateral adrenalectomy.^2^ Nonspecific clinical manifestations (fatigue, weight loss, hypotension, hyponatremia, hyperpigmentation) often delay diagnosis, which is established by the short cosyntropin stimulation test.^1,2^ Treatment involves life-long hydrocortisone (10–20 mg/day), fludrocortisone in primary AI, and mandatory stress-dose adjustment to avoid adrenal crisis; the health-related quality of life is continuously poor despite satisfactory therapy.^1,3^

The global prevalence of AI is estimated at approximately 300 per million, primary AI is 93 to 140 per million, and secondary AI is 150 to 280 per million.^4^ AI-related hospitalization has increased by 62% and adrenal crisis hospitalization by 90%, much of which is due to a rise in the load of secondary AI due to the use of immunotherapy and exogenous glucocorticoids.^4^

Coronavirus disease 2019 (COVID-19), caused by severe acute respiratory syndrome coronavirus-2 (SARS-CoV-2), is now well established as a multisystem disorder extending far beyond the respiratory tract.^5^ In addition to pulmonary involvement, COVID-19 has been associated with cardiovascular, neurological, renal, hematologic, and endocrine complications.^6,7^ Among endocrine organs, increasing attention has been paid to the hypothalamic–pituitary–adrenal (HPA) axis, given its central role in stress responses, immune modulation, and hemodynamic stability.^8,9^ Early reviews of COVID-19–related endocrinopathy highlighted disturbances in glucose metabolism, thyroid function, pituitary regulation, and adrenal physiology, suggesting that SARS-CoV-2 infection may disrupt endocrine homeostasis through multiple pathways.^8,10^

The adrenal glands are particularly susceptible to injury during systemic infections.^11^ Their rich vascular supply, high metabolic demand, and dependence on intact microcirculation render them vulnerable to ischemia, hemorrhage, inflammation, and immune-mediated damage.^12^ Prior viral infections, including SARS-CoV-1, have been linked to adrenal pathology and a long-term low-cortisol state, providing biological plausibility for similar sequelae in COVID-19.^13^ Autopsy and imaging studies in COVID-19 have further demonstrated adrenal necrosis, microthrombi, hemorrhage, and inflammatory infiltration, supporting both structural and functional adrenal involvement.^14^

Beyond direct adrenal injury, HPA axis dysregulation is a recognized feature of acute and critical illness.^9^ Excessive cytokine release, hypothalamic or pituitary dysfunction, and altered cortisol metabolism may result in an inadequate cortisol response to physiological stress, a condition often referred to as critical illness–related corticosteroid insufficiency (CIRCI).^15^ In the context of COVID-19, this phenomenon may be compounded by the disease’s pro-inflammatory and pro-thrombotic milieu, as well as by widespread exposure to exogenous glucocorticoids during treatment.^16^

Against this background, an increasing number of clinical reports have described new-onset AI in patients with COVID-19 who had no prior history of adrenal disease.^17^ These reports span a broad clinical spectrum, including secondary (central) AI attributed to hypothalamic or pituitary dysfunction^13,18,19^ and primary AI resulting from adrenal hemorrhage, infarction, or autoimmune mechanisms.^20–22^ Several cases presented during the acute phase of COVID-19, whereas others manifested weeks to months after apparent recovery, raising concerns about post-COVID-19 adrenal dysfunction.^13,17,23^

Clinically, AI in COVID-19 is particularly challenging to recognize because its manifestations—fatigue, hypotension, hyponatremia, shock, and neurocognitive symptoms—overlap substantially with features of severe COVID-19 and post-acute COVID-19 syndrome.^24^ Nevertheless, many reported cases demonstrated rapid clinical improvement following glucocorticoid replacement, underscoring the importance of timely diagnosis.^13,18^ Conversely, failure to recognize AI may result in life-threatening adrenal crisis, particularly in critically ill or post-COVID patients.^24^

Despite growing interest, the current literature remains fragmented and heterogeneous. Most available evidence consists of case reports and small observational studies, with substantial variability in diagnostic criteria, cortisol cut-off values, timing of hormonal assessment, and use of dynamic testing.^17,18^ Moreover, while some prospective data suggest preserved adrenal function in selected cohorts of patients with long COVID,^25^ these findings contrast with reports of clinically significant AI in other post-COVID settings. As a result, the actual burden, mechanisms, and clinical relevance of COVID-19–associated AI remain uncertain.

Given these uncertainties, systematically reviewing the available evidence can clarify clinical features, diagnostic strategies, and the mechanisms underlying COVID-19–associated AI in patients without prior adrenal disease. Synthesis is necessary to guide clinical care and highlight areas needing further study. The primary objective of this systematic review is to summarize and critically appraise the existing literature on new-onset AI associated with COVID-19 in patients without prior adrenal disease. Secondary objectives are to describe the types and timing of AI reported, examine the diagnostic methods used across studies, and synthesize the proposed pathophysiological mechanisms underlying adrenal and HPA-axis dysfunction in COVID-19.

In this review, “adrenal insufficiency” refers to biochemically and/or clinically documented primary or secondary adrenal failure, characterized by inadequate cortisol production with inappropriate ACTH responses, and requiring glucocorticoid replacement. Conditions consistent with a transient stress-related low-cortisol state or CIRCI, without confirmatory endocrine testing, were analyzed separately and interpreted with caution.

## 2. METHODS

### 2.1 Study design and reporting standards

This study was conducted as a systematic literature review to identify and synthesize evidence on AI in patients with COVID-19 who do not have preexisting adrenal disease. The review was designed and reported in accordance with the Preferred Reporting Items for Systematic Reviews and Meta-Analyses (PRISMA) 2020 guidelines. The protocol for this systematic review was registered in the PROSPERO International Prospective Register of Systematic Reviews (registration No.: CRD420251275141).

### 2.2 Eligibility criteria

Studies were eligible for inclusion if they reported original data on patients with laboratory-confirmed COVID-19 infection and described new-onset AI or low cortisol state in individuals without a prior diagnosis of adrenal disease. Eligible study designs included observational studies (cross-sectional, cohort, or case–control) and case reports and case series. Only articles published in peer-reviewed journals and available in the English language were considered for inclusion.

Studies were excluded if they included patients with preexisting AI or congenital adrenal disorders, were animal or in vitro studies, or consisted of editorials, commentaries, or other publications without original patient-level data. Reports describing adrenal dysfunction exclusively in relation to COVID-19 vaccination were also excluded. In addition, studies lacking sufficient clinical or biochemical information to support a diagnosis of AI were excluded.

### 2.3 Information sources

A comprehensive literature search was conducted across the following electronic databases: PubMed/MEDLINE, Scopus, and Web of Science (Core Collection). The search encompassed all studies published in the database from January 30, 2020, to December 28, 2025. In addition, the reference lists of all included articles were manually screened to identify further relevant studies that may not have been captured through the electronic database search.

### 2.4 Search strategy

The search strategy combined controlled vocabulary terms and free-text keywords related to COVID-19 and AI. Boolean operators (“AND” and “OR”) were used to optimize sensitivity. The PubMed/MEDLINE search strategy included combinations of the following terms: (“COVID-19” OR “SARS-CoV-2”) AND (“adrenal insufficiency” OR “hypocortisolism” OR “hypothalamic–pituitary–adrenal axis” OR “HPA axis” OR “ACTH deficiency” OR “central adrenal insufficiency” OR “primary adrenal insufficiency” OR “adrenal hemorrhage” OR “adrenal infarction”). Equivalent search strategies were adapted for Scopus and Web of Science, in accordance with each database’s indexing and syntax.

### 2.5 Study selection

**Figure 1.**
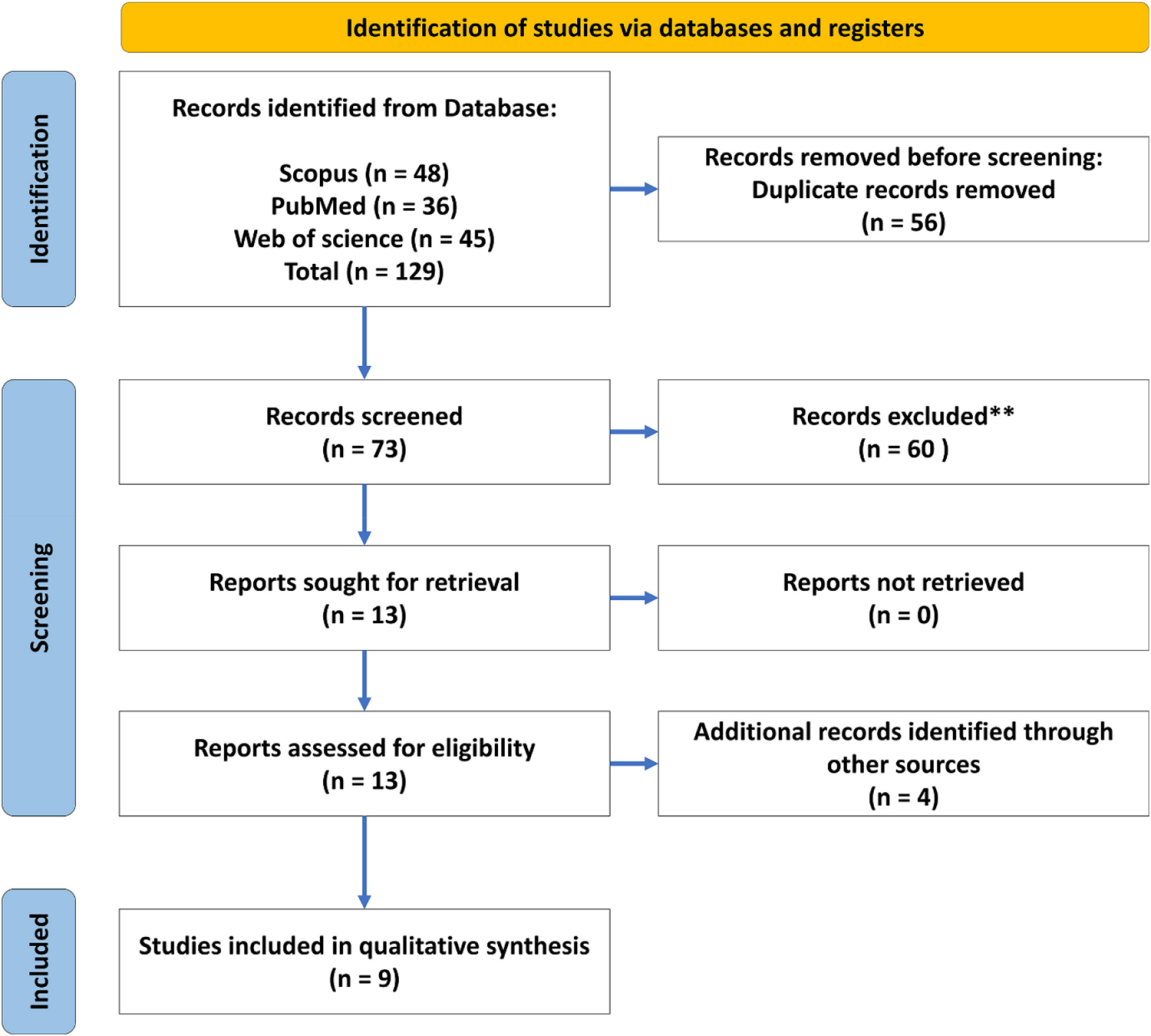
PRISMA 2020 flow diagram illustrating the study selection process for the systematic review of new-onset adrenal insufficiency in COVID-19 patients without preexisting adrenal disease.

All records retrieved from the database searches were imported into reference management software, and duplicates were removed (Figure 1). Two reviewers independently screened titles and abstracts for eligibility. Full-text articles were subsequently assessed for inclusion. Disagreements between reviewers were resolved through discussion and, when necessary, consultation with a third reviewer.

### 2.6 Data extraction

Data were independently extracted by two reviewers using a standardized data extraction form to ensure consistency and accuracy. Extracted variables included the first author and year of publication, country, study design, and study setting, patient demographics (age and sex), COVID-19 severity, diagnostic methods used to assess adrenal function, type of AI (primary or secondary), timing of diagnosis (during acute infection or post-COVID period), and clinical outcomes and management strategies. Any discrepancies between reviewers during data extraction were resolved through discussion and consensus. Where reported, cortisol thresholds and use of dynamic testing were extracted; however, no uniform diagnostic cut-off was applied across studies.

Since there is variability in diagnostic methods, the extracted data were examined in light of the level of diagnostic confirmation. Cases were conceptually divided into biochemically verified AI and case presentations that were consistent with likely CIRCI, with no definitive biochemical testing. This differentiation defines the analysis of the synthesized evidence.

### 2.7 Risk of bias assessment

Given the predominance of descriptive study designs among the included literature, methodological quality was assessed using validated tools tailored to study type. The Joanna Briggs Institute (JBI) Critical Appraisal Checklists were applied to case reports and case series, while the Newcastle–Ottawa Scale (NOS) was used to evaluate observational studies. For cross-sectional studies, the NOS was applied using a modified version (modified criteria), as recommended in prior methodological literature. Risk-of-bias assessments were conducted independently by two reviewers, with any disagreements resolved through discussion and consensus.

### 2.8 Data synthesis

Due to substantial heterogeneity in study designs, diagnostic criteria, and outcome reporting, quantitative meta-analysis was not feasible. Therefore, the findings were synthesized narratively, with studies systematically grouped according to study design, type of AI (primary versus secondary), and timing of clinical presentation, distinguishing between acute COVID-19–related cases and post-COVID manifestations. This structured qualitative approach enabled the identification of recurrent clinical patterns, the comparison of diagnostic strategies, and the integration of proposed pathophysiological mechanisms underlying adrenal dysfunction associated with COVID-19.

## 3. RESULTS

### 3.1 Study selection

The systematic search identified 129 records. After removing duplicates, 56 articles were screened based on their titles and abstracts. Of these, 41 were excluded, and 13 full-text articles were assessed for eligibility (Table 1). Ultimately, nine primary studies met the inclusion criteria and were included in the qualitative synthesis. Due to substantial heterogeneity in study designs and outcomes, quantitative meta-analysis was not performed. Articles involving patients with preexisting AI were excluded from qualitative synthesis. Accordingly, the report by Eguchi^26^ was excluded from the final analysis and is referenced only for contextual discussion.

### 3.2 Contextual literature

Narrative reviews and expert opinion articles were excluded from the qualitative synthesis and were not considered primary evidence. These articles were cited selectively to support background information, mechanistic interpretation, and clinical context where appropriate. Cortisol cutoff values and diagnostic algorithms varied across studies, reflecting differences in assay platforms, clinical contexts, and study designs.

### 3.3 Study characteristics

The characteristics of the included articles are summarized in Table 1. The studies were published between 2021 and 2025 and originated from Asia, Europe, and South America, reflecting global distribution. Most reports were single-patient case reports, while only two studies^9,23^ employed an observational design with a defined denominator. Patient ages ranged from pediatric to older adults, with a predominance of male patients among reported cases. COVID-19 severity varied from mild infection to critical illness, although AI was frequently diagnosed after the acute phase, particularly in the post-COVID or convalescent period. AI was diagnosed using morning serum cortisol measurements, often supplemented by plasma ACTH levels and, in selected cases, ACTH stimulation testing. Both primary (due to adrenal hemorrhage, infarction, or autoimmune mechanisms) and secondary (central) AI were reported, with the central low-cortisol state occurring more frequently. Clinical outcomes ranged from hemodynamic instability and adrenal crisis to fatigue, hypotension, and neurocognitive symptoms, with most cases demonstrating clinical improvement following glucocorticoid replacement therapy.

### 3.4 Risk of bias assessment

Risk-of-bias assessment was conducted exclusively for the primary studies included in the qualitative synthesis, in accordance with established methodological standards (Table 2). Narrative reviews^6,8,10,27^ and expert opinion articles were not subjected to formal quality assessment, as they do not report original patient-level data and therefore fall outside the scope of standardized risk-of-bias tools. Primary studies were evaluated using validated instruments appropriate to their study design to ensure consistency and transparency. This approach allowed for a focused appraisal of methodological quality while avoiding inappropriate assessment of secondary literature.

Table 2, which indicates the risk of bias, shows the range of the strength of the methodologies used in the included reports. The studies with a low risk of bias^13,19,21,22^ were well-known for their longitudinal data, applied dynamic or confirmatory testing of the endocrine system, and clear follow-up, which gave greater certainty to the conclusions about individual cases with confirmed AI. Conversely, articles that had a moderate or high risk of bias^17,23^ did not provide any biochemical confirmation, except for clinical steroid responsiveness, which is not useful for characterizing classical AI but remains informative when discussing functional HPA-axis dysregulation in critical illness. The observational studies^18,2^8 were deemed to be at moderate risk of bias, primarily because they were cross-sectional, did not adjust for confounders, and, in one study, lacked specificity regarding diagnostic criteria. Such studies may therefore indicate a correlation but not causality, because their design does not allow for causality.

### 3.5 Main findings

This systematic review suggests that new-onset AI can occur in patients with COVID-19 without a prior history of adrenal disease, presenting during either the acute infection or the post-COVID period.^13,18–23,28^ While the overall evidence base is dominated by case reports, a consistent clinical pattern emerges: low cortisol states accompanied by hemodynamic instability and systemic symptoms that respond rapidly to glucocorticoid replacement.^13,21,22^

The included studies reported new-onset AI in temporal association with COVID-19 across a range of clinical settings and disease severities. Central (secondary) AI was the most frequently reported type, observed in the majority of biochemically confirmed cases, and was attributed to HPA axis dysfunction.^13,18,19,28^ Primary AI, resulting from bilateral adrenal infarction, hemorrhage, or autoimmune mechanisms, was documented in a smaller subset of cases.^20–22^ AI was diagnosed both during the acute phase of COVID-19 and in the post-COVID or convalescent period, with the latter being more frequently reported across the included studies.^13,19,21–23^ Most biochemically confirmed cases demonstrated clinical improvement following initiation of glucocorticoid replacement therapy.^13,19–22^ Notably, several reports lacked confirmatory endocrine testing and relied solely on clinical shock and steroid responsiveness; these presentations are more consistent with probable CIRCI or functional HPA-axis suppression rather than classical AI, and were interpreted with appropriate caution throughout the synthesis.^18,23^

## 4. DISCUSSION

This systematic review suggests that new-onset AI can occur in patients with COVID-19 with no preexisting adrenal disease, although the current evidence base is limited and heterogeneous. A key interpretation issue is that some reported cases may represent CIRCI/functional HPA-axis suppression (particularly in critically ill patients) rather than classical AI, because confirmatory testing and standardized cortisol thresholds were often not reported. Reported cases span a broad clinical spectrum, including both central (secondary) AI, likely related to HPA axis dysfunction, and primary AI, most often associated with adrenal infarction, hemorrhage, or immune-mediated mechanisms. Adrenal dysfunction has been observed during the acute phase of COVID-19 as well as in the post-COVID period, highlighting its relevance beyond critical illness.^25^

It is important to acknowledge that the primary data source for this review consists of case reports and small observational studies. While such a design does not allow for a quantitative estimation of the incidence of new-onset AI following COVID-19, it serves as a valuable source for the initial description of a novel clinical phenomenon, hypothesis generation, and raising clinical awareness. The accumulated data consistently indicate that this condition, although likely rare, can occur across various clinical scenarios and carries significant consequences. Therefore, a key objective for future research must be the conduct of prospective cohort studies with well-defined populations and standardized diagnostic protocols to establish accurate estimates of prevalence, natural history, and risk factors.

The reported cases represent a pathophysiological continuum. At one end lies transient HPA-axis suppression during acute illness—commonly termed CIRCI—presumably mediated by cytokine-driven dysregulation. At the opposite end is irreversible adrenal damage from infarction, hemorrhage, or autoimmune destruction, resulting in permanent AI. Although both manifest as hypocortisolism, they differ fundamentally in natural history, post-acute management requirements, and the necessity for lifelong hormone replacement. Distinguishing between these entities through appropriate convalescent testing is therefore essential for accurate interpretation of the literature and clinical decision-making.

Multiple, potentially overlapping pathophysiological mechanisms may underlie AI in COVID-19. SARS-CoV-2 gains cellular entry via angiotensin-converting enzyme 2 (ACE2) receptors, which are expressed in the adrenal glands, pituitary, and hypothalamus, providing a biologically plausible basis for direct viral injury and immune-mediated inflammation of HPA-axis tissues. Autopsy studies have demonstrated adrenal necrosis, microthrombosis, and inflammatory infiltration, providing structural evidence for adrenal dysfunction.^14^ In parallel, several clinical reports and observational findings suggest central AI, likely resulting from viral hypophysitis, cytokine-driven suppression of corticotropin release, or transient hypothalamic dysfunction, consistent with observations of low or inappropriately normal adrenocorticotropic hormone levels.^13,18,19,28^ The prothrombotic state characteristic of severe COVID-19 may further increase the risk of bilateral adrenal infarction or hemorrhage, a mechanism clearly supported by imaging-confirmed cases of primary AI.^22^ In critically ill individuals, COVID-19 may also precipitate CIRCI, wherein cortisol production or action is insufficient to meet physiological stress demands despite the absence of overt adrenal destruction.^15,17,23^ Additionally, iatrogenic and immune-triggered mechanisms may contribute: exogenous glucocorticoid therapy during acute infection can unmask latent adrenal vulnerability, whereas post-infectious immune dysregulation may trigger autoimmune adrenalitis in genetically susceptible patients.^24,25^

Publication Bias and External Validity Considerations. The current evidence base is inherently susceptible to publication and selection bias, as clinically severe or atypical presentations are disproportionately reported. This may inflate the perceived clinical significance of post-COVID-19 AI. Moreover, the included studies predominantly feature hospitalized patients with moderate-to-severe disease, limiting generalizability to ambulatory or mild infections. Our conclusions are therefore most appropriately applied to higher-acuity populations, and extrapolation to community-managed COVID-19 should be undertaken with caution.

Diagnostic Heterogeneity and Interpretative Challenges. Diagnostic thresholds for AI varied substantially across the included studies. Morning serum cortisol cutoff values used to define AI ranged from <5 µg/dL to <10 µg/dL, and reference ranges varied by assay methodology and laboratory standards. Furthermore, ACTH stimulation testing was applied inconsistently, and in several reports, the diagnosis was inferred from basal cortisol levels or from clinical response to glucocorticoids alone. This heterogeneity limits cross-study comparability and precludes reliable incidence estimation, underscoring the need for standardized endocrine diagnostic criteria in future COVID-19–related adrenal research. Furthermore, the lack of standardized cortisol and ACTH immunoassays across studies introduces an additional layer of interpretative uncertainty, as different platforms vary in analytical sensitivity, specificity, and calibration. Consequently, absolute hormone values and diagnostic thresholds are not directly comparable. Moving forward, uniform diagnostic guidelines are needed. In stable post-COVID patients presenting with symptoms suggestive of AI, a formal workup including morning cortisol, ACTH, and, when indicated, ACTH stimulation testing should be considered, rather than applied as a routine screening measure in all post-COVID individuals. In critically ill patients, where dynamic testing is often impractical, the consensus CIRCI criteria, complemented by prudent clinical judgment, should be applied.

A further interpretive challenge comes up in acute-phase cases where corticosteroid therapy was needed for severe COVID-19.^29^ In these cases, clinical improvement may show anti-inflammatory effects instead of replacement activity. This limitation is especially important for cases labeled as probable CIRCI, where there was no biochemical confirmation.

From a clinical perspective, these findings underscore the importance of maintaining a high index of suspicion for AI in COVID-19 patients presenting with unexplained hypotension, shock, persistent fatigue, hyponatremia, or prolonged post-COVID symptoms.^30^ Early recognition and timely initiation of glucocorticoid replacement therapy are associated with rapid clinical improvement in reported cases and may be life-saving in severe presentations.^31^ However, routine screening of all COVID-19 survivors is not supported by current evidence, and targeted evaluation based on clinical context remains the recommended approach.^32^

From a research standpoint, the predominance of case reports and small observational studies limits the ability to estimate incidence or establish causality.^33^ Well-designed prospective studies with standardized hormonal assessments, consistent diagnostic criteria, and longitudinal follow-up are needed to clarify the actual burden, natural history, and long-term outcomes of COVID-19–associated adrenal dysfunction. Future research should also aim to distinguish transient HPA-axis suppression from persistent AI and to define subgroups that may benefit from targeted screening.

### 4.1 Strengths and limitations

This review provides a comprehensive synthesis of published evidence on COVID-19–related AI. However, conclusions are limited by heterogeneous study designs, the predominance of case reports, and the absence of extensive prospective studies. The true incidence and long-term outcomes of adrenal dysfunction following COVID-19 remain uncertain.

### 4.2 Future directions

Prospective multicenter studies with standardized hormonal testing protocols and longitudinal follow-up are urgently required to better define the burden and natural history of adrenal dysfunction associated with COVID-19. Such studies are essential for accurately determining the incidence of AI following SARS-CoV-2 infection, distinguishing transient HPA axis suppression from persistent or progressive AI, and identifying patient- and disease-related factors associated with recovery or long-term dysfunction. In addition, robust prospective data are needed to establish evidence-based screening strategies in post-COVID clinics, enabling targeted hormonal evaluation of high-risk individuals while avoiding unnecessary testing in asymptomatic patients.

## 5. CONCLUSION

In summary, AI has been reported as an infrequently reported but clinically significant endocrine manifestation temporally associated with COVID-19. Awareness of this entity is essential to prevent diagnostic delays and adverse outcomes. At the same time, further high-quality evidence is required to guide screening, management, and follow-up strategies in both acute and post-COVID care settings.

Overall, dysfunction of the HPA axis, including both established AI and likely CIRCI, has been reported to occur in temporal correlation with COVID-19. The existing data is characterized by diverse diagnostic criteria. Further research needs to be standardized to assess hormones and provide a clear picture of the epidemiology, natural history, and clinical importance of post-COVID-19 HPA-axis disorders.

## ACKNOWLEDGMENTS

The author(s) have no acknowledgements to declare.

## DISCLOSURE OF AI USE

No generative artificial intelligence tools were used in the conception, design, data collection, analysis, interpretation, or writing of this manuscript. The authors affirm that all content was produced entirely by the authors without AI assistance.

## FUNDING

This research received no specific grant from any funding agency in the public, commercial, or not-for-profit sectors.

## CONFLICT OF INTEREST

The author(s) declare that there is no conflict of interest.

## ETHICAL APPROVAL

Not applicable—systematic review of published studies.

## AUTHOR CONTRIBUTIONS

IF: Conceptualization, methodology, writing – original draft. SMA: Investigation, resources, visualization, writing – review, and editing. AMM: Data curation, formal analysis, writing – review and editing. STA: Supervision, validation, writing – review, and editing. NMM: Investigation, data curation, validation, writing – review, and editing. AT: Investigation, data curation, validation, writing – review, and editing.

## DATA AVAILABILITY STATEMENT

All data are included in the manuscript.

## Figures and Tables

**Table 1.** Characteristics of studies included in systematic review.

Author, year (Reference)	Country	Study design	Study setting	Sample size (n)	Age (years)	Sex (% male)	COVID-19 severity	Diagnostic method for adrenal insufficiency	Type of adrenal insufficiency	Timing of diagnosis	Main outcomes reported
Ach et al. 2024^28^	Tunisian	Cross-sectional observational study	Hospital	64 (32 with Long COVID; 32 recovered)	Mean ± SD: 42.9 ± 13.8 (G1: 43.3 ± 14.3; G2: 42.6 ± 13.5)	Overall: ~20.3% (G1: 12.5%; G2: 28.1%)	Statistically comparable between groups (P = .624); spectrum from asymptomatic to critical	Serum cortisol ± ACTH	Central/secondary (suspected)	Acute/post-COVID	New-onset adrenal insufficiency reported after COVID-19
Chua and Chua, 2021^13^	Singapore	Case report	Hospital	1	47	100%	Mild	Morning cortisol, ACTH, ACTH stimulation test	Secondary (central)	Post-COVID	Delayed low cortisol state responsive to hydrocortisone
Das et al. 2021^18^	India	Cross-sectional observational study	Hospital/ICU	84	42 ± 15	~52%	Mild–critical	Morning serum cortisol ± ACTH	Predominantly central	Acute COVID-19	Prevalence of low cortisol state and association with severity
Flokas et al. 2022^20^	Greece	Case report (pediatric MIS-C)	Hospital	1	7	0%	Severe	Serum cortisol, ACTH	Primary autoimmune	Acute COVID-19	MIS-C-associated adrenal insufficiency
Hashim et al. 2021^17^	India	Case report	Hospital	1	62	100%	Severe	Clinical shock + steroid responsiveness (no confirmatory endocrine testing reported)	Probable CIRCI/functional HPA-axis suppression	Post-COVID	Steroid-responsive shock due to adrenal insufficiency
Katikar, 2021^23^	India	Letter/case report	Hospital	1	62	100%	Severe	Clinical suspicion ± cortisol (insufficient endocrine confirmation)	Probable CIRCI/functional HPA-axis suppression	Post-COVID	Adrenal insufficiency presenting as shock
Kayhan et al. 2024^21^	Turkey	Case report	Endocrinology clinic	1	31	0%	Mild	Serum cortisol, ACTH, adrenal CT	Primary adrenal insufficiency	Post-COVID	Autoimmune primary adrenal insufficiency after COVID-19
Machado et al. 2022^22^	Brazil	Case report	Tertiary hospital	1	46	0%	Moderate	Cortisol, ACTH, CT/MRI	Primary adrenal insufficiency	Post-COVID	Bilateral adrenal infarction due to COVID-19
Yamasaki et al. 2024^19^	Japan	Case report	Hospital	1	67	100%	Moderate–severe	Serum cortisol, ACTH	Central adrenal insufficiency	Post-COVID	HPA-axis dysfunction following COVID-19

NR, Data not reported in the original publication; NA, Not applicable. Due to heterogeneity in study designs and predominance of case reports, a qualitative synthesis was performed.

**Table 2.** Risk of bias assessment of included studies.

Author, year (Reference)	Study design	Risk of bias tool	Overall risk of bias	Justification
Ach et al. 2024^28^	Cross-sectional study	NOS	Moderate	Cross-sectional design without longitudinal follow-up; limited adjustment for confounding variables; heterogeneity in cortisol assessment and absence of standardized diagnostic thresholds.
Das et al. 2021^18^	Cross-sectional study	NOS	Moderate	Cross-sectional design; incomplete dynamic endocrine testing in all participants; limited control for confounders; absence of longitudinal follow-up.
Chua and Chua, 2021^13^	Case report	JBI	Low	Comprehensive hormonal testing, including ACTH assessment; well-documented clinical course and response to treatment.
Flokas et al. 2022^20^	Case report	JBI	Moderate	Single-case design; adrenal dysfunction assessed during acute inflammatory MIS-C state, which may influence cortisol interpretation; absence of longitudinal reassessment.
Hashim et al. 2021^17^	Case report	JBI	Moderate	Diagnosis inferred primarily from clinical presentation and steroid responsiveness; limited biochemical confirmation; absence of dynamic testing and follow-up.
Katikar, 2021^23^	Case report/Letter	JBI	High	Incomplete biochemical documentation; absence of ACTH measurement or stimulation testing; diagnosis largely based on clinical course and steroid response; limited methodological detail.
Kayhan et al. 2024^21^	Case report	JBI	Low	Detailed biochemical evaluation and imaging confirming primary adrenal insufficiency; clear diagnostic methodology and management documentation.
Machado et al. 2022^22^	Case report	JBI	Low	Comprehensive biochemical and radiological confirmation of adrenal infarction; well-documented clinical course.
Yamasaki et al. 2024^19^	Case report	JBI	Low	Biochemical confirmation of ACTH deficiency with appropriate endocrine evaluation; clear diagnostic methodology; limitation primarily related to short follow-up duration.
